# Neuro-Oncology of Women (NOW): Special Considerations Regarding Management of Women of Reproductive Age with Glioma

**DOI:** 10.1007/s11912-026-01748-9

**Published:** 2026-03-26

**Authors:** Alexandra Simpson Farrukh, Terri Lynn Woodard, Jennifer L Hopp, Caitlyn Hull, Na Tosha Gatson

**Affiliations:** 1 Department of Neurosurgery, OU Health Sciences Center/Stephenson Cancer Center, 800 NE 10th Street Suite L100, Oklahoma City, OK 73104 USA; 2 Department of Gynecologic Oncology and Reproductive Medicine, The University of Texas MD Anderson Cancer Center, The University of Texas MD Anderson Cancer Center, 1155 Pressler Unit 1362, 77030 Houston, TX USA; 3 Department of Neurology, The University of Maryland Medical Center, 110 S. Paca St. 3rd Floor, Baltimore, MD 21201 USA; 4 The James Cancer Center/Stefanie Spielman Comprehensive Breast Center Advanced Practice Provider, Department of Surgery, The Ohio State University Wexner Medical Center, 1145 Olentangy River Road Columbus, Columbus, OH 43212 USA; 5 Neuroscience and Cancer Institutes, Indiana University School of Medicine, Indianapolis, USA

**Keywords:** Neuro-oncology of women (NOW), Gliomas in women, People with epilepsy of childbearing potential (PWECP), Women with cancer, Epilepsy in women, Sexual health, Fertility

## Abstract

**Purpose of Review:**

Cancer care for women is nuanced, and this is particularly true for neuro-oncology of women (NOW). A subset of women who require even more specialized care are those of reproductive age. The purpose of this review is to streamline guideline-directed care for women with gliomas, specifically those of childbearing potential.

**Recent Findings:**

No central resource for management of glioma in women exists, and previous studies show that clinicians do not always adhere to guideline-directed care of these patients and significant care gaps exist for NOW patients.

**Summary:**

NOW patients have special considerations in their cancer care including but not limited to pregnancy planning, chemotherapy counseling, management of epilepsy, fertility, and sexual well-being.

## Introduction

Females make up almost 60% patients diagnosed with brain and spinal cord tumors in adults [1]; however, recommendations on how to manage and treat the reproductive needs within this unique population are not well-organized or nuanced and are limited by the lack of data. Improved prognosis in glioma patients has led to a greater number of young, female glioma patients becoming pregnant despite their diagnosis [2]. Moreover, with the advent of target therapy improving progression-free survival in young patients with isocitrate dehydrogenase mutant (IDH-MT) gliomas [2], neuro-oncology providers will be responsible for counseling their patients more than ever on fertility-related issues.

A subset of patients who will require even more assistance are women of childbearing potential. Not only are neuro-oncologists responsible for the care of the patient, but they may also affect the health of their patients’ offspring.

The purpose this article is to guide neuro-oncology providers in recognizing and managing the unique issues affecting their female patients of child-bearing potential. For this article, we use the terms “female” and “woman/women” to describe patients who are assigned female at birth. However, it is important to note that these terms are not perfect and have broader and more inclusive meanings, specifically to the trans and nonbinary community. These patients have their own unique and underrepresented issues affecting their health that should also be explored but will not be highlighted in this article.

## Methods

This review will summarize the current evidence regarding management of glioma in NOW patients. Literature was selected by searching Pub Med for topics related to neuro-oncology of women, cancer in women, epilepsy management in women, chemotherapy guidelines in women, women’s reproductive health in cancer, and sexual health in women with cancer. Case reports for women who had received chemotherapy during pregnancy were reviewed from PubMed. In addition, medication labels from the Food and Drug Administration were reviewed, with particular focus on recommendations for pregnant and nursing mothers.

### Pregnancy Prevention

Although cancer during pregnancy is relatively uncommon, affecting 1/1000 pregnancies [3], the incidence is likely to rise in the future due to increased rates of early-onset cancers as well as the tendency for women to delay childbearing until their later reproductive years. In addition, female cancer survivors have been found to be three times more likely to have an unintended pregnancy compared to the general population, and are significantly more likely to utilize emergency contraception [4], making pre-treatment discussions about contraception vitally important. Cancer during pregnancy is a complex situation that requires delicate balancing of health and risks of the mother and the fetus [5]. 

General recommendations for discussing and selecting a contraceptive method for women with cancer as well as specific considerations with regard to women with CNS tumors have been published by the Society of Family Planning [6]. 

### Summary of Chemotherapy Recommendations

Chemotherapy administration is contraindicated during pregnancy, and women are recommended to use contraception during and after receiving chemotherapy. However, what should neuro-oncologists say to their female patients who wish to grow a family either before or after their cancer treatment has been completed? Will her chemotherapy affect her fertility in the long-term? These are complex questions with nuanced answers, and most women would benefit from a referral to an onco-fertility specialist or reproductive specialist. As neuro-oncologists, we also have a duty to inform and guide and support our patients to make the best decision for themselves and their family.

This section will summarize the recommendations for the most prescribed chemotherapies for neuro-oncology patients and will highlight specific NOW considerations. In all the discussed medications, there are no adequate data from randomized-controlled studies in pregnant or breast-feeding women (nor will there be in the future) to inform pregnancy risks. Therefore, risk is inferred from pre-clinical animal models and recommendations are generated based on pharmacokinetics.

Although teratogenicity of all chemotherapeutic and anti-cancer agents is not yet well-delineated, it is advisable to postpone pregnancy until after administration of these therapies [6]. Recommendations for timing of conception remains unclear after completion of therapy are not well standardized but should take into consideration the general health of the patient, obstetrical risks, pharmacokinetics of the treatment agent and risk for cancer recurrence. A case report has shown that pregnancy and the birth of a healthy infant can occur as early as three weeks after the last chemotherapy and even after heavy pretreatment with alkylating agents [7], however it should be noted that the long-term effects on the health of offspring exposed to chemotherapy are unknown [7]. 

### Temozolomide

Temozolomide (TMZ) is a common chemotherapy in neuro-oncology and is used for a variety of tumors including but not limited to glioblastoma, oligodendroglioma, and astrocytoma [8]. Temozolomide is a Pregnancy Category D medication, meaning that TMZ can cause fetal harm when administered to a pregnant woman [9]. It is not recommended that WCBP become pregnant while on TMZ. In addition, they should be advised to use effective contraception while on this medication as well as *for six months after the last dose*. TMZ manufacturers do not have specific recommendations regarding type of contraception [9]. Regarding lactation, it is not known if TMZ is excreted in human milk, but there is a potential harm to nursing infants.

Interestingly, there have been a handful of published case reports of pregnant mothers who received various cycles of TMZ at various doses who did not experience any perinatal or postnatal complications. The five reported cases identified by Evans et al. demonstrated no harm to mother or fetus; however, it is important to note that these patients received only a few doses of TMZ before pregnancy was discovered, and the medication was abruptly discontinued [10]. 

### Procarbazine

Procarbazine hydrochloride (procarbazine) is often used in combination with lomustine (CCNU) and vincristine for treatment of IDH-MT gliomas [8]. Procarbazine can cause fetal harm when administered to a pregnant woman, and there are case reports of malformations in the offspring of women who were exposed to procarbazine hydrochloride in combination with other antineoplastic agents during pregnancy [11, 12]. It is not recommended for women of child-bearing protentional to become pregnant and they should use contraception. However, drugs manufacturers have not commented on the time interval between chemotherapy discontinuation and contraception discontinuation. It is not recommended to breastfeed while on procarbazine [11, 12]. 

### Vorasidenib

Vorasidenib is a an FDA-approved medication indicated for IDH-MT World Health Organization (WHO) Grade 2 astrocytoma and oligodendroglioma as detected by an FDA-approved test, following surgery including biopsy, sub-total resection, or gross total resection [13]. Moreover, this medication is also being used off label for high grade IDH-MT gliomas. and will likely be studied in these populations in future trials. The prevalence of low-grade gliomas is more common in people in their twenties to forties, i.e. their reproductive years [14]. This medication poses a unique question to neuro-oncologists, as it can be a lifelong therapy (unlikely traditional chemotherapy) for patients with low-grade gliomas.

Regarding family planning, vorasidenib can cause fetal harm when administered to pregnant women, and the drug manufacturers recommend waiting at least three months since discontinuation of vorasidenib before attempting conception. They also recommend using nonhormonal contraception during and for three months after last dose, as vorasidenib may render some hormonal contraceptives ineffective [13]. 

Notably, levonorgestrel-releasing intrauterine devices (IUDs) act locally in the uterus and have a much smaller amount of systemic hormone exposure compared to oral contraceptives and are often not lumped into the traditional hormonal contraceptive class. On the other hand, the etonogestrel implant releases progesterone into systemic circulation and is metabolized through the CYP3A4 substrate [15]. Both the makers of the etonogestrel implant and vorasidenib warn concomitant use of CYP3A inducers [13, 15]. If systemic hormonal birth control cannot be avoided, the nonhormonal contraceptive methods should be used in conjunction.

The makers of vorasidenib do not recommend breastfeeding while on this medication [13]. 

### Lomustine

Lomustine (CCNU) has many applications in neuro-oncology including first-line treatment in IDH-MT gliomas. It can also be used in recurrent isocitrate dehydrogenase-wildtype (IDH-WT), IDH-MT gliomas, and ependymomas [8]. CCNU can cause fetal harm when administered to a pregnant woman. The makers of CCNU recommend using “effective” contraception during treatment and for two weeks after final dose. They also advise women to not breastfeed while on the medication and for two weeks after final dose [16]. 

### Bevacizumab

Bevacizumab is a humanized monoclonal antibody that targets vascular endothelial growth factor (VEGF) ligand and prevents binding to VEGF receptors, and is used neuro-oncology for treatment of primary gliomas as well as radiation-induced brain necrosis [8]. 

Although, this drug has not been studied specifically in pregnant glioma patients, its use in pregnancy has been reported in gynecological cancers, as bevacizumab is used in the treatment of cervical cancer. Because the VEGF pathway influences maintaining the corpus luteum and amnionic fluid regulation, bevacizumab in the pregnant can induce a pre-eclampsia-like syndrome of hypertension and proteinuria [17]. Thus, bevacizumab use is not recommended in pregnancy. Moreover, the makers of bevacizumab not only recommend contraception while on the medication, but they also recommend women continue “adequate” contraception for at least six months following the last dose [18]. 

As with many other medications, it is unknown if the medication is transferred to human breast milk, and if so, what the long-term risks to the nursing child are. The manufacturers recommend a joint decision between patient and clinician regarding breastfeeding while on bevacizumab, but published data suggest that breast milk antibodies do not enter the neonatal and infant circulation in substantial amounts [18]. 

### Vincristine

Vincristine sulfate (vincristine) is a chemotherapy used in multiple types of cancers such as leukemia, lymphoma, neuroblastoma, and Wilms tumor. In the neuro-oncology world, vincristine is more commonly utilized in pediatric populations, but for adults, it is often used in conjunction with procarbazine and lomustine in IDH-MT gliomas as well as in adult medulloblastoma regimens [8]. Vincristine can cause fetal malformations and death when administered to a pregnant woman, and the makers of vincristine recommend that women should be advised to avoid pregnancy [19]. As with all of the other chemotherapies, it is unclear if the drug is excreted in milk, but the manufacturers do not recommend nursing while on the medication.

In summary, women should utilize effective contraception prior to initiating treatment as well as during treatment, preferably contraception that does not affect the cytochrome P450. Contraception is also recommended for a period after treatment has been completed has been ceased, and the time interval depends on the clearance rate of the specific medication.

If a clinician discovers that his or her patient is pregnant, chemotherapy, regardless of agent, should be stopped. A referral to a maternal fetal medicine specialist for additional counseling and management recommendations should be offered so that women can make informed decisions about her pregnancy. It is as the discretion of the patient’s wishes whether she wishes to carry her pregnancy. It is important to note that decisions on whether or not to continue pregnancy may be dictated by state laws where the patient resides. For most patients, it is not recommended to breastfeed while on treatment given lack of safety data, but a patient and provider may pursue shared decision-making regarding risks and benefits (Table 1).

**Table 1:** Summary of chemotherapy recommendations for NOW patients

Medication Name	Interval After Last Dose Until Contraception is Recommended	Contraception Recommendations By Drug Manufacturer	Breastfeeding Considerations
TMZ	6 months	no specific recommendations	no specific recommendations, potential harm
Procarbazine	no specific recommendations	no specific recommendations	not recommended
Vorasidenib	3 months	nonhormonal methods or IUD	not recommended
CCNU	2 weeks	“effective”	wait at least 2 weeks
Bevacizumab	6 months	“adequate”	likely safe
Vincristine	no specific recommendations	nonhormonal methods or IUD	not recommended

### Treatment of Seizure and Epilepsy in NOW Patients

Approximately 35% −70% of people with brain tumors go on to develop epilepsy, and seizures are more common in low grade (WHO Grade 2) gliomas than higher grade gliomas [20]. In general, it is not recommended to offer seizure prophylaxis for patients with intracranial tumors [20]. When a NOW patient of reproductive age warrants an anti-seizure medication (ASM), there are important considerations regarding this specialized management that include safety during pregnancy, lactation, and potential interactions with chemotherapy.

The American Academy of Neurology (AAN), American Epilepsy Society (AES), and the Society of Maternal-Fetal Medicine (SMFM) revised and updated their joint guidelines in May 2024 regarding seizure management in PWECP.

If clinically feasible, it is recommended that women with epilepsy who can conceive be managed with levetiracetam, oxcarbazepine, or lamotrigine monotherapy, as these medications are associated with the lowest risk of major congenital malformations [21]. If possible, valproic acid and topiramate should be avoided in women with epilepsy given to minimize the risk of offspring being born small for gestational age [22]. Moreover, valproic acid use in pregnancy is associated with higher incidents of children with neurodevelopmental sequalae such as neural tube defects, lower IQs, and autism-spectrum disorder [21]. 

For patients with brain tumors, levetiracetam is the most well-studied and prescribed anti-seizure medication [21]. Levetiracetam has few drug interactions; can be administered intravenously or orally; has a less common mechanism of action; and can be used when other anti-seizure medications that are contra-indicated because of hepatic or cardiac co-morbidities [23]. Moreover, levetiracetam, along with oxcarbazepine and lamotrigine, have Level A associated evidence supporting their use in PWECP. In the algorithm published by the Society of Neuro-Oncology (SNO), first line therapy for a single seizure aligns with AES guidelines to use levetiracetam, oxcarbazepine, and lamotrigine [24]. 

Notably, another common ASM used by neuro-oncologists is lacosamide [24, 25]. There is more limited data in its use in pregnancy but preliminary data from retrospective analyses suggest that this medication has a favorable safety profile in pregnancy [26]. In the most recent data from the North American Pregnancy registry, no children born to the out of the 93 women on lacosamide had a major congenital malformation [27]. 

On the contrary, along with its potential teratogenic effects, valproic acid, an enzyme-inhibitor, may worsen leukopenia and thrombocytopenia caused by chemotherapy [28]. In addition, enzyme-inducing ASMs such as carbamazepine, phenytoin, phenobarbital, and primidone may decrease the efficacy of chemotherapy [24, 28]. 

With regard to ASMs and lactation, breast-feeding is encouraged and does not appear to cause any measurable long-term risks for the child [7]. This is typically encouraged as an individual choice for the family.

Furthermore, the AAN, AES, SMFM, and the United States Preventative Services Task Force (USPSTF) also recommend daily supplementation with folic acid 0.4 mg to all women on anti-seizure medications who can become pregnant [21]. Notably, this recommendation is for any woman who can become pregnant, as it is estimated that over a third of pregnancies are unplanned in the United States [22]. 

In summary, female patients with brain tumor and epilepsy should be prescribed monotherapy with levetiracetam, lamotrigine, or oxcarbazepine monotherapy, though decision-making should be appropriate for seizure and syndrome type and receive folic acid supplementation regardless of pregnancy status. It is also typically recommended that management of pregnancy and seizures in people with child-bearing potential be managed by epileptologists with specialty expertise in this area as these patients require additional ASM level monitoring, counseling in breastfeeding, and additional safety precautions after delivery (Table 2).

**Table 2:** Summary of Anti-Seizure recommendations for NOW patients

Summary of ASM Recommendations NOW Patients
Monotherapy with levetiracetam, lamotrigine, oxcarbazepine, or lacosamide, * if clinically feasible
Folic acid supplementation
Referral to epilepsy specialist for duration of pregnancy and postpartum period
Avoidance of valproic acid, carbamazepine, phenytoin, phenobarbital, and primidone if clinically feasible

*not guideline recommended but has favorable safety profile.

### Fertility and Sexual Health Concerns in NOW Patients

As long-term survival rates of young patients with cancer have improved, there has been a corresponding increase of physical and psychological quality-of-life (QoL) issues experienced by these patients [29]. In this section, we will focus on two pertinent and often under addressed QoL issues and in NOW patients of reproductive age: fertility preservation (FP) and sexual health.

### Fertility Considerations

One of the most distressing effects of cancer treatment in young women is infertility. Infertility can be due to ovarian toxicity caused by chemotherapy or radiotherapy, which can result in pre-mature ovarian failure (POF) [29]. In particular, alkylating agents are more likely to cause premature ovarian failure (POF). Many of the most common chemotherapy agents in neuro-oncology (TMZ and procarbazine) are DNA alkylating agents. Moreover, CCNU is considered highly toxic to fertility. According to the CED calculator (cyclophosphamide equivalent dose), CCNU typical dosing for gliomas is about 10,560 CED. A dose over 4000 CED is considered significantly increased risk of infertility. These patients are at a very high risk of infertility and should be prioritized for fertility preservation (FP) [30]. 

Ionizing radiation can lead to primary ovarian insufficiency (POI) as well. Ionizing radiation induced POI most often occurs when the ovaries are in the radiation field, which is usually not applicable in neuro-oncology patients. However, brain irradiation can be associated with a reduction of fertility due to a toxicity to the hypothalamic–hypophyseal axis; however, patients can still be able to become pregnant with the use of gonadotropins [31]. 

The American Society of Clinical Oncology (ASCO) recommends that healthcare providers discuss fertility risks and FP with all patients or reproductive age as early as possible, and these discussions should be documented [32, 33]. Patients who are interested in or ambivalent about FP should be referred to reproductive specialists. Fertility preservation “represents all clinical efforts to preserve gametes or reproductive tissue for future use in patients who desire genetic parenthood.” Examples include the “administration of gonadotropin-releasing hormone (GnRH) agonists, ovarian tissue cryopreservation, ovarian stimulation with cryopreservation of oocytes and/or embryos, fertility-sparing surgery and oophoropexy.” [29].

A consult with an onco-fertility or reproductive specialist should include a general health and reproductive history, assessment of the patient’s ovarian reserve, and an estimation of the patient’s risk for infertility based on her treatment plan, age and other fertility factors. She should be educated on the FP options that are available to her (as well as their risks, success rates and cost) and counseled on all potential options for family building during survivorship, including third party reproduction, such as the use of donor eggs/embryos and use of a gestational carrier [34]. Patients should also be offered follow-up after cancer treatment for reassessment of ovarian reserve and discussions on future pregnancy and family building plans.

Despite ASCO recommendations, neurooncologists often do not discuss FP with brain tumor patients [35]. There are many explanations as to why neuro-oncologists do not bring up FP to patients: such as comfort of the clinician having the discussion, the emphasis on more time-sensitive issues, limited time during appointments, concern that FP may delay treatment, functional status of the patient, cost of FP, or even appropriateness of the conversation during visits [36]. Stone et al., looked at their own institution’s referrals, and found that only 7% of their patients were appropriately referred to FP with a fertility nurse specialist despite ASCO’s recommendation [35]. Although these few statistics represent a few centers, it is reasonable to infer that many neuro-oncology providers are likely not properly adhering to ASCO and National Comprehensive Cancer Network (NCCN) recommendations regarding FP referral.

Notably, in a small cohort Lehman et al., revealed that only about half of cancer survivors were aware of the risk of infertility from their cancer treatment, and these women were more likely to be distressed about not being able to have biological children when compared to their male counterparts [35]. Furthermore, NOW patients do in fact have a significant interest in FP, especially if they had no prior children [31]. Finally, Lombard et al. shows that patients undergoing FP prior to chemotherapy and/or radiation for a glioma achieve satisfactory FP outcomes in comparison to controls, showing the importance of discussing FP more systematically prior to treatment [31]. 

In summary, NOW patients should receive a referral to an onco-fertility specialist or reproductive specialist to discuss fertility risk and options for fertility preservation as part of comprehensive cancer care. Moreover, neuro-oncologists should provide general fertility education to their patients and provide support regarding fertility and family planning regardless of hesitation.

### Additional Pregnancy Considerations

Though rare and potentially underreported, gliomas are one of the more common solid tumors found in pregnant women, after meningioma and breast cancer [1]. It has been observed that pregnant “glioma patients can experience increased tumor aggressive behaviors such as increased lesion size and associated vasogenic edema, clinical decline, seizures, and development radiographic features of tumor malignant transformation.” [1].

In 2014, Yust-Katz et al. published a retrospective chart review of 34 pregnant glioma patients and observed that all five patients with Grade 1 had stable disease during and after pregnancy. Nearly half of the patients with Grade 2 or 3 disease had disease progression [37]. Notably, these tumors were graded by the 2007 WHO criteria. This study reported that of the pregnancies taken to full-term, all children were born “healthy.” Currently, there are no expert consensus on surveillance guidelines or recommendations for safe timing and use of chemotherapy and radiation in these patients [1]. 

While the care of the pregnant glioma patient is often performed on a case-by-case basis with a multidisciplinary discussion, there are ACOG recommendations for medical complications of pregnancy that can be applied to glioma patients. For example, in a pregnant NOW patient with venous thromboembolism (VTE), the preferred treatments are continuous intravenous heparin in the acute setting with transition to low-molecular weight heparin (LMWH) for chronic management. For patients with contraindications to anticoagulation, an inferior vena cava (IVC) filter can be placed. These treatments are also safe breast-feeding infant. For the non-nursing patient, direct oral anticoagulants (DOACs) can be used [38] (Table 3).

**Table 3:** Summary of VTE recommendations for pregnant glioma patients

Summary of VTE Recommendations for Pregnant Glioma Patients
Continuous heparin in acute setting
LMWH injection in chronic setting
IVC filter
DOAC in chronic setting for non-nursing patient

### Sexual Health Considerations

Sexual health is another priority concern of young women with cancer. Some cancers, such as diffuse low-grade gliomas frequently affect young adults, and life expectancy often extends over several decades. As a result, preserving quality of life—including sexual health—is crucial. The Diagnostic and Statistical Manual of Mental Disorders, Fifth Edition (DSM-5), recognizes three distinct types of sexual dysfunction (SD) in women: female orgasmic disorder, sexual interest/arousal disorder, and genitopelvic pain/penetration disorder [39]. Unfortunately, SD is an often-overlooked sign or symptom by both patients and clinicians [40].

From an anatomical and functional level, the pituitary gland, hypothalamus, limbic system, or frontal cortex can directly affect sexual health. In addition, changes in physical appearance such as hair loss, scarring, and changes in weight from surgery or side effects from medical intervention can cause self-esteem issues, which can then cause reduced sexual confidence [40]. Moreover, ASCO and NCCN guidelines recommend regular screening of patients for sexual health side effects.

Prior cancer survivorship research on sexual wellness in women mostly comes from breast and gynecologic cancers. There remains a research and care gap for NOW patients. One group in Italy evaluated a small cohort of 46 brain tumor patients with questionnaires and found that nearly three fourths of the female patients reported disorders of sexual desire; over half reported arousal and orgasmic disorders; and half reported orgasmic disorders [41]. In another study, Lombard et al., also found that women were more likely to have symptoms of SD than men [31]. Despite the high prevalence of dysfunction, just 15% of patients were informed about possible sexual effects, with few asking for details [41]. 

Sexual health is an important part of a person’s well-being, and mental health affects physical health. Though treatment of cancer has rapidly advanced and increased survival rates, some outlooks toward cancer survivorship have not. And these issues are very pertinent to all NOW patients, especially those of reproductive age (Table 4).

**Table 4:** Summary of fertility and sexual health considerations in NOW patients

Fertility and Sexual Health Considerations in NOW Patients
Fertility preservation discussion at initial or early visits
Referral to onco-fertility or reproductive specialist
Post-cancer treatment follow-up
Regular screening for sexual health side effects

## Conclusion

As neuro-oncologists, we must learn to incorporate this education and line of questioning into our routine practices. Though a small subset of the entire cancer population, NOW patients, especially those of reproductive age, face unique, critical, and important issues that do not necessarily just affect them but their potential children as well. Possible interventions to these issues are not necessarily time-consuming or mentally challenging. They merely require thoughtful evaluation and counseling of patients, as well as adherence to already-published guidelines from academic societies (Table 5).

**Table 5:** Clinical checklist for NOW patients

Clinical Checklist for NOW Patients
Contraception discussion and referral to PCP or OBGYN
Review of chemotherapies with teratogenicity and fertility implications
Review of antiseizure medications
Folic acid supplementation
Regular sexual health questionnaires and documentation of conversations

## Key References

- Avila EK, Tobochnik S, Inati SK, et al (2024) Brain tumor-related epilepsy management: A Society for Neuro-oncology (SNO) consensus review on current management. Neuro-Oncol 26:7–24.
  - SNO has guidelines for management of tumor-related epilepsy in the general glioma population. These recommendations were then cross-referenced with AES recommendations for people of child-bearing potential to optimize care for NOW patients.
   
- Pack AM, Oskoui M, Williams Roberson S, et al (2024) Teratogenesis, Perinatal, and Neurodevelopmental Outcomes After In Utero Exposure to Antiseizure Medication: Practice Guideline From the AAN, AES, and SMFM. Neurology 102:e209279.
  - AAN, AES, and SMFM recently updated their ASM guidelines from 2017 to be more inclusive and offer more specific recommendations for women with epilepsy syndrome, reproductive status, and potential impact on the developing child. These recommendations were cross-reference with SNO recommendations above.
- Lombard A, Duffau H (2022) Sexual Dysfunction of Patients with Diffuse Low-Grade Glioma: A Qualitative Review of a Neglected Concern. Cancers 14:3025.
  - People with low grade gliomas can survive years to decades and these tumors most often occur during reproductive years. Yet there is very little literature regarding SD and its affect on quality of life in glioma patients. This article is one of few published reviews of sexual dysfunction evaluation in glioma patients and explores how SD is often overlooked and undervalued by clinicians.

## Data Availability

No datasets were generated or analysed during the current study.

## References

[1] Gatson NTN, Boccia ML, Taylor KR, Mack JKO, Fonkem E. Hormone-dependent tumors and sexuality in the neuro-oncology of women (N.O.W.): women’s brain tumors, gaps in sexuality considerations, and a need for evidence-based guidelines. Curr Oncol Rep. 2021;23:127. 10.1007/s11912-021-01115-w. 34453233 10.1007/s11912-021-01115-w

[2] Mellinghoff IK, Van Den Bent MJ, Blumenthal DT, Touat M, Peters KB, Clarke J, et al. Vorasidenib in IDH1- or IDH2-mutant low-grade glioma. N Engl J Med. 2023;389:589–601. 10.1056/NEJMoa2304194. 37272516 10.1056/NEJMoa2304194 PMC11445763

[3] Cottreau CM, Dashevsky I, Andrade SE, Li D-K, Nekhlyudov L, Raebel MA, et al. Pregnancy-associated cancer: a U.S. population-based study. J Womens Health. 2019;28:250–7. 10.1089/jwh.2018.6962. 10.1089/jwh.2018.6962 PMC6390809 30307780

[4] Medica ACO, Stark SS, Hadnott TN, Dietz AC, Romero SAD, Natarajan L, et al. Use of emergency contraception among female young adult cancer survivors. Fertil Steril. 2018;109:1114-1120.e1. 10.1016/j.fertnstert.2018.02.136. 29935646 10.1016/j.fertnstert.2018.02.136 PMC6020163

[5] Sorouri K, Loren AW, Amant F, Partridge AH. Patient-centered care in the management of cancer during pregnancy. Am Soc Clin Oncol Educ Book. 2023;e100037. 10.1200/EDBK_100037. 37220323 10.1200/EDBK_100037

[6] Batur P, Brant A, McCourt C, Schwarz EB. Society of family planning clinical recommendation: contraceptive considerations for individuals with cancer and cancer survivors part 3 – skin, blood, gastrointestinal, liver, lung, central nervous system, and other cancers: joint with the Society of Gynecologic Oncology. Contraception. 2025;147:110869. 10.1016/j.contraception.2025.110869. 40328522 10.1016/j.contraception.2025.110869

[7] Van Westrhenen A, Senders JT, Martin E, DiRisio AC, Broekman MLD. Clinical challenges of glioma and pregnancy: a systematic review. J Neurooncol. 2018;139:1–11. 10.1007/s11060-018-2851-3. 29623596 10.1007/s11060-018-2851-3 PMC6061223

[8] Nabors LB, Holdhoff M, Peters KB. NCCN Guidelines Index Table of Contents Discussion. 2025.

[9] TEMODAR. (temozolomide) Label.

[10] Evans AC, Nelson MB, Dhall G. Pregnancy in a patient with a malignant brain tumor taking Temozolomide: Case Report and Review of the Literature. 10.1177/1043454214563414 25576318

[11] MATULANE. ^®^(procarbazine hydrochloride) Capsules.

[12] Matulane_PI_Aug_2018.

[13] label.

[14] Cao J, Yan W, Zhan Z, Hong X, Yan H. Epidemiology and risk stratification of low-grade gliomas in the United States, 2004–2019: a competing-risk regression model for survival analysis. Front Oncol. 2023;13:1079597. 10.3389/fonc.2023.1079597. 36937393 10.3389/fonc.2023.1079597 PMC10014976

[15] NEXPLANON. (etonogestrel implant) Label.

[16] GLEOSTINE. (lomustine) capsules, for oral use.

[17] Korenaga T-RK, Tewari KS. Gynecologic cancer in pregnancy. Gynecol Oncol. 2020;157:799–809. 10.1016/j.ygyno.2020.03.015. 32268951 10.1016/j.ygyno.2020.03.015 PMC7380448

[18] AVASTIN^®^ (bevacizumab) Label.

[19] vincristine.

[20] Walbert T, Harrison RA, Schiff D, Avila EK, Chen M, Kandula P, et al. SNO and EANO practice guideline update: anticonvulsant prophylaxis in patients with newly diagnosed brain tumors. Neuro-Oncology. 2021;23:1835–44. 10.1093/neuonc/noab152. 34174071 10.1093/neuonc/noab152 PMC8563323

[21] Pack AM, Oskoui M, Williams Roberson S, Donley DK, French J, Gerard EE, et al. Teratogenesis, Perinatal, and Neurodevelopmental Outcomes After In Utero Exposure to Antiseizure Medication: Practice Guideline From the AAN, AES, and SMFM. Neurology. 2024;102:e209279. 10.1212/WNL.0000000000209279. 38748979 10.1212/WNL.0000000000209279 PMC11175651

[22] Rossen L, Hamilton EB, Abma J, E CW, Beresovsky V, Resendez A, et al. Updated Methodology to Estimate Overall and Unintended Pregnancy Rates in the United States [Internet]. National Center for Health Statistics (U.S.); 2023. 10.15620/cdc:124395.

[23] Howard P, Remi J, Remi C, Charlesworth S, Whalley H, Bhatia R, et al. Levetiracetam. J Pain Symptom Manage. 2018;56:645–9. 10.1016/j.jpainsymman.2018.07.012. 30036676 10.1016/j.jpainsymman.2018.07.012

[24] Avila EK, Tobochnik S, Inati SK, Koekkoek JAF, McKhann GM, Riviello JJ, et al. Brain tumor-related epilepsy management: a Society for Neuro-oncology (SNO) consensus review on current management. Neuro-Oncology. 2024;26:7–24. 10.1093/neuonc/noad154. 37699031 10.1093/neuonc/noad154 PMC10768995

[25] Perucca P, Bourikas D, Voinescu PE, Vadlamudi L, Chellun D, Kumke T, et al. Lacosamide and pregnancy: data from spontaneous and solicited reports. Epilepsia. 2024;65:1275–84. 10.1111/epi.17924. 38411300 10.1111/epi.17924

[26] Puris G, Chetrit A, Katorza E. Fetal safety in MRI during pregnancy: a comprehensive review. Diagnostics. 2025;15:208. 10.3390/diagnostics15020208. 39857092 10.3390/diagnostics15020208 PMC11765095

[27] Latest Data. – AED 2023.

[28] Kanner AM, Bicchi MM. Antiseizure medications for adults with epilepsy: a review. JAMA. 2022;327:1269. 10.1001/jama.2022.3880. 35380580 10.1001/jama.2022.3880

[29] Pereira N, Schattman GL. Fertility preservation and sexual health after cancer therapy. J Oncol Pract. 2017;13:643–51. 10.1200/JOP.2017.023705. 28809602 10.1200/JOP.2017.023705

[30] Green DM, Nolan VG, Goodman PJ, Whitton JA, Srivastava D, Leisenring WM, et al. The cyclophosphamide equivalent dose as an approach for quantifying alkylating agent exposure: a report from the childhood cancer survivor study. Pediatr Blood Cancer. 2014;61:53–67. 10.1002/pbc.24679. 23940101 10.1002/pbc.24679 PMC3933293

[31] Lombard A, Duffau H. Sexual dysfunction of patients with diffuse low-grade glioma: a qualitative review of a neglected concern. Cancers. 2022;14:3025. 10.3390/cancers14123025. 35740690 10.3390/cancers14123025 PMC9221288

[32] Oktay K, Harvey BE, Partridge AH, Quinn GP, Reinecke J, Taylor HS, et al. Fertility preservation in patients with cancer: ASCO clinical practice guideline update. J Clin Oncol. 2018;36:1994–2001. 10.1200/JCO.2018.78.1914. 29620997 10.1200/JCO.2018.78.1914

[33] Su HI, Lacchetti C, Letourneau J, Partridge AH, Qamar R, Quinn GP, et al. Fertility preservation in people with cancer: ASCO guideline update. J Clin Oncol. 2025;43:1488–515. 10.1200/JCO-24-02782. 40106739 10.1200/JCO-24-02782

[34] Gupta D, Singh S, Shukla S, Shrivastava S. Oncofertility: treatment options from bench to bedside. Cancer Pathog Ther. 2023;1:284–9. 10.1016/j.cpt.2023.05.001. 38327602 10.1016/j.cpt.2023.05.001 PMC10846294

[35] Stone JB, Kelvin JF, DeAngelis LM. Fertility preservation in primary brain tumor patients. Neuro-Oncol Pract. 2017;4:40–5. 10.1093/nop/npw005. 10.1093/nop/npw005 PMC5815629 29479452

[36] Gatson N, Milbourne NT, Ornelas A, Nevel S, Boccia KS. ML. The impact of brain tumor location and treatment on sexual function: Implications for clinical practice. Neuropsychol Psychosoc Found Neuro-Oncol [Internet]. Elsevier; 2024 [cited 2025 Jan 15]. pp. 299–306. 10.1016/B978-0-443-15663-2.00039-0

[37] Yust-Katz S, De Groot JF, Liu D, Wu J, Yuan Y, Anderson MD, et al. Pregnancy and glial brain tumors. Neuro Oncol. 2014;16:1289–94. 10.1093/neuonc/nou019. 24615863 10.1093/neuonc/nou019 PMC4136891

[38] Kalaitzopoulos DR, Panagopoulos A, Samant S, Ghalib N, Kadillari J, Daniilidis A, et al. Management of venous thromboembolism in pregnancy. Thromb Res. 2022;211:106–13. 10.1016/j.thromres.2022.02.002. 35149395 10.1016/j.thromres.2022.02.002

[39] Candy B, Chi Y, Graham-Wisener L, Jones L, King M, Lanceley A, et al. Interventions for sexual dysfunction following treatments for cancer in women. Cochrane Pain, palliative and supportive care group. Editor Cochrane Database Syst Rev [Internet]. 2016. 10.1002/14651858.CD005540.pub3. [cited 2025 Mar 5];2022. 10.1002/14651858.CD005540.pub3 PMC9301918 26830050

[40] Gatson NTN, Szabo RJ, Tadipatri R, Lee GW, Mach AA. Unpacking the psychological, cognitive, and psycho-pharmacologic burdens of neuro-oncology. Neuropsychol Psychosoc Found Neuro-Oncol [Internet]. Elsevier; 2024 [cited 2025 Jan 15]. pp. 327–82. 10.1016/B978-0-443-15663-2.00038-9

[41] Finocchiaro CY, Petruzzi A, Fedeli G, Vistalli MG, Innocenti A, Silvani A, et al. Hidden reality: sexual sphere in brain tumor patients. Psychol Health Med. 2017;22:370–80. 10.1080/13548506.2016.1210176. 27431202 10.1080/13548506.2016.1210176

